# Synthesis, Characterization and Antitumour Activity of Metal Complexes of 5-Carboxy-2-Thiouracil

**DOI:** 10.1155/MBD.1998.35

**Published:** 1998

**Authors:** Udai P. Singh, Sudha Singh, Sukh Mahendra Singh

**Affiliations:** 1 Department of Chemistry University of Roorkee Roorkee 247 667 India; 2 Department of Chemistry Banaras Hindu University Varanasi 221 005 India; 3 School of Biotechnology Banaras Hindu University Varanasi 221 005 India

## Abstract

Metal complexes of 5-carboxy-2-thiouracil with Mn(ll), Co(ll), Ni(ll), Cu(ll), Zn(ll) and Cd(ll) ions were synthesized, characterized, and subjected to a screening system for evaluation of antitumour activity against Sarcoma-180 (S-180) tumour cells. The complexes were characterized by elemental analysis, infrared, electronic spectra, room temperature magnetic measurements and powder X-ray diffraction. The antitumour activity results indicate that some complexes have antitumour activity both in vivo and in vitro against S-180 tumour cells.


### SYNTHESIS, CHARACTERIZATION AND ANTITUMOUR ACTIVITY OF METAL COMPLEXES OF 5-CARBOXY-2-THIOURACIL

Udai P. Singh*[1] , Sudha Singh[2a] and Sukh Mahendra Singh[2b]

[1] Department of Chemistry, University of Roorkee, Roorkee-247 667 (U.P.), India
[2a] Department of Chemistry [2b] School of Biotechnology
Banaras Hindu University, Varanasi-221 005, India

**Abstract**

Metal complexes of 5-carboxy-2-thiouracil with Mn(II), Co(II), Ni(II), Cu(II), Zn(II) and Cd(II) ions were synthesized, characterized, and subjected to a screening system for evaluation of antitumour activity against Sarcoma-180 (S-180) tumour cells. The complexes were characterized by elemental analysis, infrared, electronic spectra, room temperature magnetic measurements and powder X-ray diffraction. The antitumour activity results indicate that some complexes have antitumour activity both in vivo and in vitro against S-180 tumour cells.

**Introduction**

5-Carboxy-2-thiouracil (5CTU) (Fig. 1) containing nitrogen, sulphur and oxygen as donor atoms, plays an important

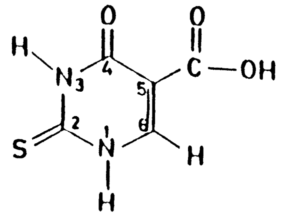

Figure 1: 5-Carboxy-2-thiouracil role in anticancer and antiviral activities [1]. The bacteriostatic or bacteriocidal activities of 5CTU and scanty information on its metal complexes [2] as antitumour agent encouraged to undertake a systematic study of its metal complexes and to test the antitumour activity of synthesized compounds. The present paper describes the structural as well as the antitumour activity studies of Mn(II),Co(II), Ni(II), Cu(II), Zn(II) and Cd(II) complexes with 5CTU.

**Results and Discussion**

The satisfactory elemental analyses have been obtained for all compounds. They display 1:1 stoichiometry. All the complexes are coloured except Zn(II) and Cd(II). They are insoluble in almost all common organic solvents except Mn(II) complex which is sparingly soluble in dimethylsulfoxide on heating.

**Infrared spectra**

Table 1 shows some important infrared absorption bands of 5CTU and its complexes. Complexation can be expected to lead the shifts in the carboxylate, thioamide and amide band positions in the spectra of the complexes. The characteristic bands of the coordinated water molecule are present in the lower region of the spectra. The position of $\nu(COO)_{asym}$ (1660 $cm^{-1}$) and $\nu(COO)_{sym}$ (1450 $cm^{-1}$) bands are shifted after complexation. The shift of $\nu(COO)_{asym}$ band towards higher wave numbers and $\nu(COO)_{sym}$ band towards lower wave numbers suggest the unsymmetrical bonding of the carboxylate group and it acts as monodentate ligand towards one metal ion. The thioamide bands also show shifting in the spectra of complexes whereas $\delta N_1$-H, $\delta N_3$-H bands do not exhibit any shift suggesting that $N_1$-H and $N_3$-H groups do not interact with metal ions.

Thus the binding of 5CTU with metal ions is suggested through COO and C=S groups which have been confirmed on the basis of the presence of νM-0 and νM-S bands in the lower region of spectra [3].

**Table 1** Infrared spectral data of 5CTU and its complexes

| Band assignment | 5CTU | Mn(II) | Co(II) | Ni(II) | Cu(II) | Zn(II) | Cd(II) |
|---|---|---|---|---|---|---|---|
| vO-H | - | 3470w | 3340w | 3480m | 3375w | 3380w | 3310w |
| vN-H | 3130m | 3150m | 3145m,b | 3080w | 3120w | 3120w | 3120w |
| vC=O,vC=C | 1708s | 1735s | 1730s | 1732m | 1730s | 1735s | 1738s |
| | 1690s | 1710w | 1708m | 1712m | 1710w | 1710w | 1658w |
| $v(COO)_{asym}$ | 1660s | 1690s | 1695s | 1700m | 1685s | 1690m | 1705s |
| v C=S | 1565s | 1585m | 1584s | 1580m | 1585m. | 1580w | 1582s |
| | 1175s | 1205s | 1205s | 1205s | 1208m | 1210s | 1195s |
| | 1155w | - | - | - | 1195w | - | 1185w |
| $\delta N_{(1)}$-H | 1505w | 1502m | 1500s | 1502m | 1502s | 1505m | 1502m |
| $v(COO)_{sym}$ | 1450s | 1410s | 1420s | 1415s | 1405s | 1425m | 1408s |
| $\delta N_{(3)}$-H | 1408s | 1405s | 1418s | 1412s | 1420w | 1408w | 1405w |
| $vM\text{-}O_{aquo}$ | - | 375w | 430m | 405m,b | 468s | 376w | 350w |
| vM-S | - | 267m | 264m | 267m | 265m | 266m | 267m |
| vM-O | - | 235w | 240w | 238w | 230w | 240w | 235w |

The infrared spectra of the complexes show bands at 1410, 1310, 1030 and 820 $cm^{-1}$ in Mn(II)-5CTU; at 1438, 1295, 1020 and 815 $cm^{-1}$ in Co(II)-5CTU; at 1400, 1310, 1039 and 820 $cm^{-1}$ in Ni(II)-5CTU; at 1390, 1320, 1040 and 824 $cm^{-1}$ in Cu(II)-5CTU; at 1410, 1310, 1043 and 820 $cm^{-1}$ in Zn(II)-5CTU and at 1410, 1316, 1042 and 820 $cm^{-1}$ in Cd(II)-5CTU complexes which confirm the monodentate behaviour of coordinated nitrate [4].

### Electronic spectra and Magnetic moment

The magnetic moment of Mn (II)-5CTU complex is 5.75 B.M. and its electronic spectrum exhibits bands at 357 nm, 375nm and 460 nm which correspond to $^6A_{1g} \rightarrow {}^4A_{1g}$ (F), $^6A_{1g} \rightarrow {}^4T_{2g}$ (D) and $^6A_{1g} \rightarrow {}^4A_{1g}$, $^4E_d$(G) transitions respectively in an idealized octahedral geometry [5,6]. The spectrum of Co(II) compound is characterized by two main bands at 415 nm and 895 nm which may be assigned to $^4T_{1g}$ (F)$\rightarrow$ $^4T_{1g}$(P) and $^4T_{1g}$ (F)$\rightarrow$ $^4T_{2g}$(F) transitions respectively. The band due to $^4T_{1g}$(F)$\rightarrow$ $^4A_{2g}$ (F) transitions appeared at 1220 nm. The magnetic moment 4.80 B.M. is within the limits of the octahedral region for Co(II) compound [7]. The magnetic moment for Ni(II)-5CTU complex is 3.59 B.M., the occurrence of d-d transition bands at 444 nm $^3A_{2g}$(F)$\rightarrow$ $^3T_{1g}$(P), 580 nm $^3A_{2g}$(F) $\rightarrow$ $^3T_{1g}$(F) and at 900nm $^3A_{2g}$(F)$\rightarrow$ $^3T_{2g}$(F) in the spectrum of Ni(II) complex can be attributed to an octahedral geometry for the Ni(II) complex [8]. Cu(II)-5CTU compound shows magnetic moment value 1.88 B.M., a distorted octahedral structure is suggested. The electronic spectrum is characterized by a band at 720 nm which may be due to $^2E_l \rightarrow {}^2T_{2g}$(D) transition [6].

### Powder X-ray diffraction studies

X-ray diffraction data (not shown) of the complexes were indexed according to the method of Ito [9]. The indexing pattern yields lattice constants a=14.35,b=4.58 and c=3.53 Å for Co(II)-5CTU; a=14.39, b=5.05 and c=4.81 Å for Ni(II)-5CTU; a=8.61, b=6.55 and c=3.80 Å for Cu(II)-5CTU; a=14.56, b=5.04 and c=4.87 Å for Zn(II)-5CTU; a=8.46, b=6.36 and c=5.26 Å for Cd(II)-5CTU and suggest orthorhombic symmetry for all these complexes while Mn(II)-5CTU complex is amorphous.

On the basis of above studies, the structure of the complexes may be proposed as below:

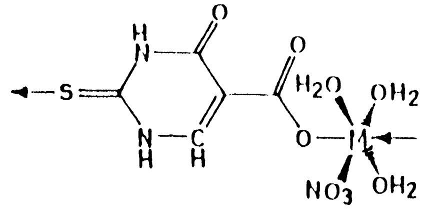

M(II)=Mn(II), Co(II), Ni(II), Cu(II), Zn(II) and Cd(II)

**Antitumour activity against Sarcoma-180**

The ligand 5CTU has the antitumour activity with T/C value 115 at the dose of 25.0 mg/kg body weight. Among the transition metal complexes only Co(II), Ni(II) and Cu(II) complexes have significant antitumour activity (Table 2). With Co(II)-5CTU and Cu(II)-5CTU treatment at the dose of 12.5 and 25.0 mg/kg body weight respectively all mice survived beyond six months.

Further it was found that none of the test compounds were toxic up to the dose of 50.0 mg/kg body weight except the complex containing Cd(II) ion (as observed by body weight change upt to six days of drug treatment). The compound Cd(II)-5CTU was found to be toxic even at the dose of 2.5 mg/kg body weight.

**Table 2** In vivo antitumour activities of 5CTU and its metal complexes against Sarcoma-180

| Compounds | Dosage ip injection mg/kg body weight[c] | Mean life span of non-survivors T/C[a] | No. of mice surviving >6 months[b] | T/C% |
|---|---|---|---|---|
| 5CTU | 12.5 | 32/40 | - | 80.0 |
| | 25.0 | 46/40 | - | 115.0 |
| $Mn(5CTU)(NO_3).3H_2O$ | 12.5 | 40/40 | - | 100.0 |
| | 25.0 | 26/40 | - | 65.0 |
| $Co(5CTU)(NO_3).3H_2O$ | 12.5 | All alive | 6 (100) | - |
| | 25.0 | 32/40 | - | 80.0 |
| $Ni(5CTU)(NO_3).3H_2O$ | 12.5 | 48/40 | - | 120.0 |
| | 25.0 | 34/40 | - | 85.0 |
| $Cu(5CTU)(NO_3).3H_2O$ | 12.5 | 38/40 | - | 95.0 |
| | 25.0 | All alive | 6(100) | - |
| $Zn(5CTU)(NO_3).3H_2O$ | 12.5 | 36/40 | - | 90.0 |
| | 25.0 | 30/40 | - | 75.0 |
| $Cd(5CTU)(NO_3).3H_2O$ | 12.5 | 2/40 | - | 5.0 |
| | 25.0 | 2/40 | - | 5.0 |

In each set of experiment six mice were used. T/C = 6/6, T= tumoured, C = control. (a) in calculating average survival time mice surviving >6 months were not included. (b) number of parenthesis indicates percentage of mice surviving six months or more. (c) single injection of the reported dose was given.

The ligand 5CTU and its complexes were also tested for their inhibitory effect on $^3H$-thymidine, $^3H$-uridine and $^3H$-leucine incorporation in S-180 tumour cells in vitro. Most of the compounds that caused inhibition of $^3H$-thymidine, $^3H$-uridine and $^3H$-leucine incorporation in S-180 tumour cells, also showed antitumour activity in vivo (Table 3a, 3b, 3c).

**Table 3a.** Percentage inhibition of $^3H$-thymidine incorporation in tumour cells in vitro *

| Compound | Dose | | |
|---|---|---|---|
| | 5 μg/ml | 10μg/ml | 20 μg/ml |
| $Co(5CTU)(NO_3).3H_2O$ | 37 | 18 | - |
| $Ni(5CTU)(NO_3).3H_2O$ | 15 | 77 | 84 |
| $Cu(5CTU)(NO_3).3H_2O$ | 21 | 50 | 68 |

Thus it is concluded from the resluts obtained in the present study that all the complexes have octahedral structure. Regarding the antitumour activity study, the ligand 5CTU and its complexes with Co(II), Ni(II) and

**Table 3b.** Percentage inhibition of $^3$H-uridine incorporation in tumour cells in vitro *

| Compound | Dose | | |
|---|---|---|---|
| | 5 μg/ml | 10μg/ml | 20 μg/ml |
| Co(5CTU) $(NO_3).3H_2O$ | 20 | 21 | 45 |
| Ni(5CTU) $(NO_3).3H_2O$ | - | - | - |
| Cu(5CTU) $(NO_3).3H_2O$ | 32 | 28 | 21 |

**Table 3c.** Percentage inhibition of $^3$H-leucine incorporation in tumour cells in vitro *

| Compound | Dose | | |
|---|---|---|---|
| | 5 μg/ml | 10μg/ml | 20 μg/ml |
| Co(5CTU) $(NO_3).3H_2O$ | 49 | 52 | 55 |
| Ni(5CTU) $(NO_3).3H_2O$ | - | - | - |
| Cu(5CTU) $(NO_3).3H_2O$ | 52 | 63 | 65 |

* These tables show the results obtained for the compounds which show significant inhibition.

Cu(II) ions have antitumour activity. The mechanism of antitumour action of these compounds is not well known. It is propable from the results obtained in the present study that these compounds may be effective antitumour agents due to their inhibitory action on the replication of DNA, synthesis of RNA and protein in tumour cells.

### Experimental

### Method of Synthesis

1m mole of hydrated metal nitrates were dissolved in a mixture of 35ml ethylalcohol and 15 ml triethylorthoformate by refluxing for about 10hrs. Then, 1m mole of 5CTU was added and the resultant mixture was refluxed for 20-25 days after which the volume of the reaction mixture was reduced to about one third of its original volume and the precipitation was initiated by adding ether. The solid complexes were separated by filtration, washed several times with ethanol, finally with ether and dried at 50-55$^0$c.

The metal ions were determined volumetrically after dissolving the complexes in dilute nitric acid [10]. Carbon, hydrogen, and nitrogen were analyzed with a Perkin-Elmer model 240C elemental analyzer. The infrared spectra were obtained in Nujol on a Perkin-Elmer model 783 spectrophotometer in the 4000-200 cm$^{-1}$ region. The electronic spectra of the complexes were recorded in Nujol with a UV/VIS-168A Shimadzu spectrophotometer at room temperature. The room temperature (298K) magnetic susceptibility measurements were made on Cahn Faraday magnetic susceptibility balance. X-ray powder diffraction data of the complexes were obtained on a Philips X-ray diffractometer PW1710 using CuKα radiation. Indexing of X-ray powder lines was done by Ito's method [9].

Antitumour activity of the ligand 5CTU and its synthesized metal complexes were tested against Sarcoma-180 (S-180) tumour system both in vivo and in vitro according to the method described earlier [11]. Therapeutic effectiveness of each compound against tumour bearing mice was assessed from the T/C percentage which was calculated as follows:

$$T/C\% = \frac{\text{Mean life span of treated mice*}}{\text{Mean life span of untreated mice}} \times 100$$

* excluding tumour free survivors.

A T/C value of 115 indicates significant activity whereas ≥ 125 indicates that complex is worthy of testing in other tumour systems [12].

**Acknowledgements**
The authors are grateful to the Head, Department of Chemistry, B.H.U., for spectral measurements.

**References**

1. Ali, A.; Livingstone, S.E. Coord. Chem. Rev. 13, 102, 1974.
2. Doody, Br. E.; Trucee, E.R.; Scruggs, R.; Li, N.C. J. Inorg. Nucl. Chem.., 28, 833, 1966.
3. Bellamy, L.J. The Infrared Spectra of Complex Molecule, Methuen London, pp. 344, 1958.
4. (a) Pandey, G.S.; Nigam, P.C.; Agrawala, U.C. J. Inorg. Nucl. Chem., 39, 1877, 1977. (b) Nakamoto, K. Infrared Spectra of Inorganic and Coordination Compounds, Wiley Interscience, New York, 2nd edition, 1970. (c) Behrens, N.B.; Goodgame, D.L.; Warke, Z. Inorg. Chim. Acta, 31, 257, 1978.
5. Figgis, B. N. Introduction to Ligand Field, Wiley Eastern, Bangalore, Ist edition, 1966.
6. Lever, A. B. P. Inorganic Electronic Spectroscopy, Elsevier, Amsterdam, 1968.
7. Palmer, R.A., ; Piper, T.S. Inorg. Chem., 5, 864, 1966.
8. Martin, J.W.L.; Timmons, J.H.; Martell, A.E.; Willis, C.J. Inorg. Chem., 19, 2328, 1980.
9. Azaroff, L.V. Elements of X-ray Crystallography, New York, 1968.
10. Flaschka, H.A. EDTA Titration, Pergamon, London, 1964.
11. Singh, U.P.; Ghose, R.; Ghose, A.K.; Singh, R.K.; Sodhi, A.; Geeta, B. Indian J. Cancer Chemotherapy, 13, 45, 1991.
12. Livingstone, S.E. Proceedings of the 20th International Conference on Coordination Chemistry, Calcutta, India, pp. 141, 1980.

**Received: August 8, 1997 - Accepted: August 25, 1997 -**
**Received in revised camera-ready format: January 14, 1998**

